# Supplementary material for: Self-Adaptation of Pseudomonas fluorescens Biofilms to Hydrodynamic Stress
Source: Front Microbiol. 2021 Jan 12;11:588884. doi: 10.3389/fmicb.2020.588884 (PMC7835673; doi:10.3389/fmicb.2020.588884)
Supplement: Supplementary file 1 [file Data_Sheet_1.pdf]

Self-adaptation of *Pseudomonas fluorescens* biofilms to hydrodynamic stress

Josué Jara, Francisco Alarcón, Ajay K. Monnappa, José Ignacio Santos, Valentino Bianco, Pin Nie, Massimo Pica Ciamarra, Ángeles Canales, Luis Dinis, Iván López-Montero, Chantal Valeriani and Belén Orgaz

April 19, 2020

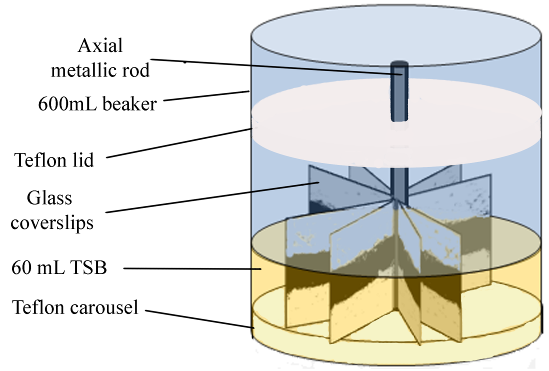

Figure S1: System set up for the biofilm development.

1 Population Dynamics models

1.1 Basic model

The initial goal of our model is to capture the general behavior of biofilm formation regarding cell counts, that is, a first exponential growth phase followed by saturation and decay of populations. For that we build on the logistic model from population dynamics or ecology without any spatial description of the experiment. We model population density and compare directly with experiments. Considering a cell number density ρ_b, the logistic model of growth is

$$\dot{\rho}_b = r\left(1 - \frac{\rho_b}{K}\right)\rho_b \quad (1)$$

which already has an initial exponential growth controlled by the growth rate r (approximately the inverse of division time). It saturates at $\rho_b^{st} = K$ where K is known as the *carrying capacity* of the medium for this reason. This value is a steady state and thus the logistic model shows no decay.

Our basic assumption is that there is a substance (oxygen, nutrients, etc...) at concentration c that is consumed by the bacteria and when scarce reduces both the growth rate and the carrying capacity. It is known that a limiting nutrient may affect growth rate following Monod's equation [1], which in the limit of low concentration (which is a reasonable one since we expect this factor to be limiting) can be reduced to a linear dependence. For the carrying capacity, it is also accepted that a limiting factor may control the maximum sustainable size of a population in the environment [2]. We suggest this particular dependence:

$$K \propto c^2 \quad (2)$$

$$r \propto c \quad (3)$$

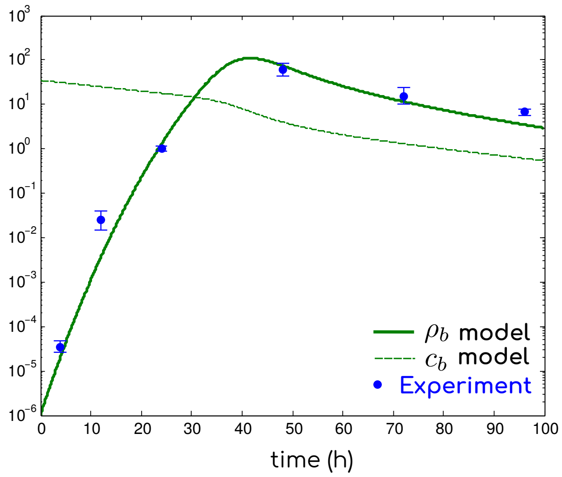

Figure S2: Fit of experimental normalized data (blue circles with error bars) with basic population dynamics model. All data normalized by the value at 24h. Biofilm cell density ρ_b (green thick line) and nutrient concentration c (green dashed line) are obtained numerically solving evolution equations (4) and (5) from model with parameters: $\alpha = 100$, $\beta = 0.023$, $\delta = 6 \times 10^{-4}$ and $b = 0.028$.

which gives then

$$\dot{\rho}_b = \beta c \rho_b - \frac{\rho_b^2}{\alpha c} \tag{4}$$

with parameters α and β constants not depending on ρ_b or c. Evolution equation for nutrient concentration is given by

$$\dot{c} = -\delta \rho_b c - bc \tag{5}$$

with δ the feeding rate and b the autodegradation rate, also supposed constant.

All parameters β, α, δ, b as well as initial concentration $c(0)$ and population $\rho_b(0)$ can be fitted to match the experimental data as shown in Figure 2. Interestingly, the 6h growth rate from the fit is $r = \beta c(6) \approx 0.68\,\mathrm{h}^{-1}$, giving a duplication time of $T \approx \frac{1}{r}\log(2) \simeq 1.0\,\mathrm{h}$, in agreement to what is expected for *P. fluorescens*.

As discussed in the main text, agitation conditions during growth (shaking or static) have a crucial impact on population evolution. Static data can also be approximately reproduced with a different set of parameters. However, it is difficult to imagine a mechanism that greatly affects every parameter. Another possible solution is to refine the model with some added complexity. This is explained in the next section.

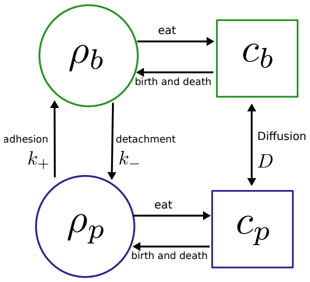

variable	description
$\rho_b(t)$	bacterial density in **biofilm**
$\rho_p(t)$	bacterial density in **plankton**
$c_b(t)$	nutrient concentration in **biofilm**
$c_p(t)$	nutrient concentration in **plankton**

Figure 3: Schematic view of model variables and interactions. For example, biofilm bacteria feed on nutrients inside biofilm, therefore affecting the concentration of nutrients in the biofilm. On the other hand, nutrient concentration modifies birth and death rates.

1.2 Model with 2 species and 2 concentration variables

The rationale behind this more complex model is that shaking may increase nutrient diffusion, bacterial transport or both. We therefore designed a model with planktonic and biofilm bacteria described by $\rho_p(t)$ and $\rho_b(t)$. Cells can migrate from one population to the other with constant (in time) rates k_+ and k_-. Also concentration of nutrients c_p and c_b may differ in both sections and thus the nutrients may diffuse. However, our model is still without explicit spatial coordinates. Evolution equations are:

$$\dot{\rho}_b = \beta_b c_b \rho_b - \frac{\rho_b^2}{\alpha_b c_b} + k_+ \rho_p - k_- \rho_b \tag{6}$$

$$\dot{\rho}_p = \beta_p c_p \rho_p - \frac{\rho_p^2}{\alpha_p c_p} - k_+ \rho_p + k_- \rho_b \tag{7}$$

$$\dot{c}_b = -\delta_b c_b \rho_b - b c_b + D(c_p - c_b) \tag{8}$$

$$\dot{c}_p = -\delta_p c_p \rho_p - b c_p - D(c_p - c_b) \tag{9}$$

D parameter controls diffusion of nutrients from biofilm to bulk and viceversa. k_+ and k_- represent transition rates from bulk to biofilm and viceversa.

Figure 3 schematically represents the variables in the model and the different interactions.

For parameter values that imply well mixed nutrients and high attachment rate, i.e., D, k_+ sufficiently high, and low detachment rate, the model provides a biofilm population density that approximately follows the measured values in shaking conditions, as depicted in Figure 3A in main text. The parameter values for the 2 species model with shaking conditions have been set to $\beta_b = 0.023$,$\beta_p = 0.030$,$\alpha_b = 80$,$\alpha_p = 100$,$\delta_b = \delta_p = 6 \times 10^{-4}$,$b = 0.028$,$c_{b0} = c_{p0} = 35$,$\rho_{b0} = 0$, $\rho_{p0} = 8 \times 10^{-7}$,$k_+ = 1$,$D = 1$ and $k_- = 10^{-3}$.

Keeping all other parameters equal, simply modifying the transition rates and diffusion constant has a strong enough impact on biofilm population to

explain the difference between shaking and static conditions. As discussed in the main text, just decreasing k_+ and D to simulate less mixing and increasing the detachment rate k_- to simulate low adhesion, is enough to reproduce approximately the static experimental data, as shown in Figure 3B in the main text.

1.3. Planktonic population

These population models can also fit the behavior of planktonic population. Figure S3 shows the data from an independent experiment where planktonic population was recorded. The data shows the same behavior of exponential growth, saturation and decay and has been fitted using the basic model described in section 3.1.

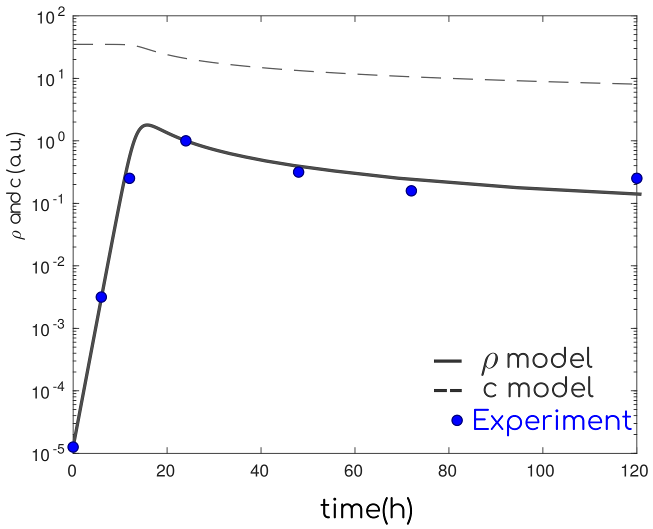

Figure S3: Planktonic population under shaking, normalized to its value at 24h. Blue dots: experimental data, gray lines: basic model results for bacterial density ρ_p and nutrient concentration c.

2 Simulations Details

We have numerically investigated the growth of bacteria colonies, using an hybrid approach involving molecular dynamics and Monte Carlo simulations. Our numerical approach has been inspired by previous ones [3, 4, 5], which we have extended to incorporate at the same time motility, reproduction and matrix induced attraction between the bacteria.

2.1 Mechanical description of a bacterium

We model a bacterium as a rod made of a sequence of $n = 7$ particles on a line. The number of particles can be varied, but we have found that $n = 7$ is a good compromise between computational cost and realistic description. An angle-bending interaction between every three consecutive particles forces the particles to be on a line. All results obtained are in the limit of large stiffness, where the bending of the rod is negligible. Particles of different bacteria interact via a LJ potential, as detailed later, so that each particle has a linear size l_p, and each bacterium is a rod with length $L(0) = l_p + (n-1)l_b(0)$, where l_b is the distance between two consecutive particles of a bacterium, which can vary during a simulation as a bacterium grow. The initial value of l_b is chosen such that $L(0) \simeq 1.8\,\mu\text{m}$, with aspect ratio $L(0)/l_p \simeq 3$, as appropriate for bacteria.

An unconstrained bacterium reproduces in a time $t_{\text{rep}} = 60\text{min}$, which is the time at which its length doubles. We model the growth of the bacterium making the equilibrium bond length a function of time. For a bacterium born at time $t_b = 0$, which is expected to reproduce at time t_{rep}, we fix $l_b(t) = l_b(0) + (l_e - l_0)\frac{t}{t_{\text{rep}}}$, and $l_b(t) = l_e$ for $t > t_{\text{rep}}$. Here l_e is chosen such that $L(t_{\text{rep}}) = 2L(0)$. When in a crowded environment, the size of a bacterium also depends on the physical forces that the other bacteria exerts on it. When the size of a bacterium becomes larger than $2L(0)$, then the bacterium reproduces. The reproduction is modeled by replacing a single bacterium of length $2L(0)$ with two bacteria of length $L(0)$, which occupy the same volume as the initial one. Note that the bacteria have a polarity fixed by their preferred direction of motion. The polarity is randomized when a bacteria reproduces.

2.2 Interaction between bacteria

The interaction between two bacteria results from the interaction of their constituent particles. The interaction is a shifted LJ potential, $U(r) = 4\epsilon\left[\left(\frac{\sigma}{r}\right)^{12} - \left(\frac{\sigma}{r}^{6}\right)\right] + c$ for $r < r_{\text{cut}}$, 0 for $r > r_{\text{cut}}$, where the constant c is chosen such that $U(r_{\text{cut}}) = 0$. We have used $r_{\text{cut}} = 2.5\sigma$.

2.3 Dynamics

We assume the bacteria to be in the overdamped regime, and therefore apply to each particle making up a bacterium a viscous for $-\gamma v$ proportional to its velocity. Here γ is a viscous friction coefficient. We assume the bacteria to perform a run and tumble motion. During a 'run' period, whose duration is a random number drawn from an exponential distribution with time constant t_{run}, we apply to the particles making a bacterium a force $F = v_{\text{run}}/\gamma$, where v_{run} is the velocity of the particles in the running state. This force acts along the polarity direction of the bacterium. During a 'tumble' period, whose duration is a random number drawn from an exponential distribution t_{tumble} min, we apply to the bacterium a torque T, which fixes a rotational velocity. The equation of motion is solved with a Verlet algorithm with timestep 5×10^{-3} s.

The dynamical properties of a bacterium depend on the species, mutant, as well as on the experimental condition. Here we have considered parameter values able to reproduce the mean square displacement curves of Ref. [6], conducted in the early stage of formation of *P. aeruginosa* biofilms. Specifically, $t_{\text{run}} = 3\,\text{min}$, $v_{\text{run}} = 0.12\,\mu\text{m}\cdot\text{s}^{-1}$, $t_{\text{tumble}} = 0.5\,\text{min}$. With these parameters, the diffusion coefficient results $D \simeq 0.7\,\mu\text{m}^2\cdot\text{s}^{-1}$.

3 Shear simulations of the biofilm

We adopt a dissipative particle dynamics (DPD) method, to model and simulate the formation of biofilm and its reaction to external stimuli. The DPD is a particle-based computational method adapt to describe systems at the mesoscale, that recently has been efficiently used to simulate biofilms [7, 8, 9]. Each DPD particle represents a cluster of molecules, whose level of coarsening depends on the chosen parameters.

Here following we will refer to the beads forming the bacteria, the polymers and the solvent with the subscribes b, p and s respectively; we will also express the quantitative in internal units, clarifying at the end their mapping to real units. We model the bacterium as a bacillus (rod-shaped) composed by several beads, each of diameter σ, held together via harmonic interactions (Figure 5). The central part of a bacterium is shaped as an empty cylinder of length $l_b/\sigma = 6$, while the extremes of the bacteria are shaped as spherical caps of radius $R_{\text{ext}}/\sigma = 4$. Both the central and extreme parts have an external radius $R_{\text{ext}}/\sigma = 4$ and internal radius $R_{\text{int}}/\sigma = 2$, i.e. the bacterium membrane is formed by three layers of particles.

The bonding interaction between bacterium's beads is given by an harmonic potential $K_{b,b}(r_{b,b} - \sigma)$, where $r_{b,b}$ is the distance between two bonded beads. This interaction acts among all the beads that, in the initial configuration of the bacterium (Figure S4), are first neighbor along the X, Y and Z directions. The polymers are composed by single chains of length l_p, each one formed by l_p/σ beads, bonded consecutively with an harmonic potential $K_{p,p}(r_{p,p} - \sigma)$, being $r_{p,p}$ the distance between two consecutive beads along the polymer backbone. The solvent is represented by a gas of DPD particles, and fill all the empty space left in the simulation box. We choose $K_{p,p} = K_{b,b} = 30$, and fix $k_B T = 1$, being k_B the Boltzmann constant and T the temperature.

All the non bonded interactions are modeled according to the DPD force field. In particular, the force on a particle of type i with position $\vec{r}_i$ and velocity $\vec{v}_i$ due to the interaction with a particle of type j with the position $\vec{r}_j$ and velocity $\vec{v}_j$ is given by the sum of three terms: the conservative term $\vec{F}^C_{i,j} \equiv A_{i,j} w(r_{i,j}) \hat{r}_{i,j}$; the dissipative term $\vec{F}^D_{i,j} \equiv -\gamma w^2(r_{i,j})\,(\hat{r}_{i,j} \cdot \hat{v_{i,j}})\,\hat{r}_{i,j}$; the random term $\vec{F}^R_{i,j} \equiv w(r_{i,j}) \left(\frac{2k_B T\gamma}{dt}\right)^{1/2} \alpha \hat{r}_{i,j}$. In the previous expression i) $r_{i,j} \equiv |\vec{r}_{i,j}|$, with $\vec{r}_{i,j} \equiv \vec{r}_i - \vec{r}_j$; ii) $\hat{r}_{i,j} \equiv \vec{r}_{i,j}/r_{i,j}$; iii) $\hat{v}_{i,j} \equiv \vec{v}_{i,j}/v_{i,j}$ with $\vec{v}_{i,j} \equiv \vec{v}_i - \vec{v}_j$ and $v_{i,j} \equiv |\vec{v}_{i,j}|$; iv) $w(r_{i,j}) = 1 - r_{i,j}/r_c$ is a weighting factor varying from 0 to 1; v)

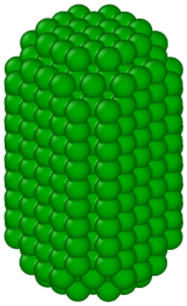

Figure S4: Proposed model for a bacterium

α is a Gaussian random number with zero mean and unit variance; vi) $A_{i,j}$ is the amplitude of the conservative force between the particles i and j (larger values of $A_{i,j}$ involve stronger repulsion and correspond to a higher level of coarse-graining); vii) $dt = 0.05$ is the integration time of the equation of motion. All the terms vanishes for $r_{i,j} > r_c$ being r_c the cutoff distance. We fix $r_c = 2\sigma$ for all the DPD interactions. In such a way the polymer-polymer crossing is strongly inhibited being the $p-p$ equilibrium distance half of the cutoff distance for the repulsive interaction. We choose all the interaction with the solvent particles to be $A_{s,s} = A_{s,p} = A_{s,b} = 25$. This values has been proven to reproduce correctly the solvent properties of water [10]. For the $p-p$, $b-b$ and $p-b$ interactions we choose larger values, $A_{p,p} = A_{b,b} = A_{p,b} = 30$, which guarantee the hydration of the bacteria and polymers (fixing $A_{p,p} = A_{b,b} = A_{p,b} = 25$ would result in a collapse of all the bacteria and polymers into a unique globular cluster). Simulations are performed with LAMMPS, with a simulation box of size $L = 32$, and fixing $\sigma = 0.5$.

According to the experimental setup, we simulate: i) a system with 184 bacteria, whose volume occupies $\sim 70\%$ of the simulation box (static growth of the biofilm), with 80 polymers, each commposed by 100 beads, whose total volume is $\sim 10\%$, and 35000 solvent particles; ii) a system with 105 bacteria, whose volume occupies $\sim 40\%$ of the simulation box (growth of the biofilm under external stress), with 240 polymers occupying a total volume of $\sim 30\%$, and 45000 solvent particles. The number of solvent particles is chosen to have a numerical density (considering the available volume for the solvent) larger than 3, to guarantee the correctness of the solvent hydrodynamics [10].

We take $kT = 4.11 \times 10^{-21}$ J as the characteristic energy scale in our simulation. Also, we can fix our length scale to the size of the bacteria, which correspond to $\sim 1.5\,\mu\text{m}$, and in our simulation it extent to $l_b + 2R_{\text{ext}} = 7$, which result

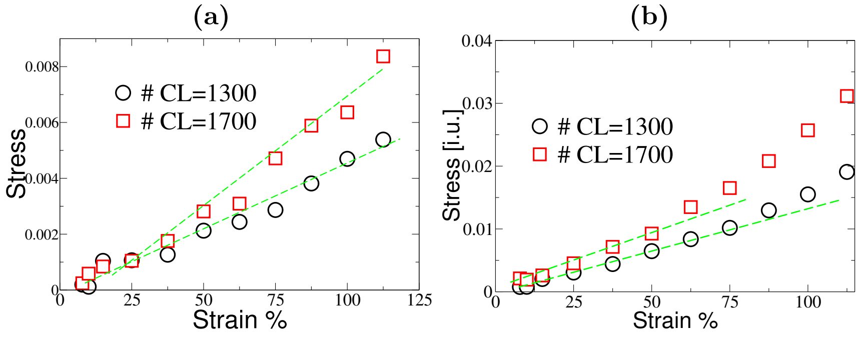

Figure S5: (a) In-phase stress σ' as function of the amplitude of the box deformation (stress-strain curve) for biofilm grown in static conditions (bacteria fills the $\sim 70\%$ of the volume), for systems with 1300 and 1700 crosslinks (CL). σ' is linear in all the range of applied deformations. Data are expresed in internal units. (b) Stress-strain curves for biofilm grown under external stimuli. The linear regime is observed for deformations up to $\sim 50\%$.

in a unit length corresponding to $l_u \sim 200\,\mathrm{nm}$ (and $\sigma \sim 100\,\mathrm{nm}$). Note that with this unit length the total size of a single polymer Pel in the experiments would correspond to a single bead. For this reason we assume that our polymer chains initially inserted in the system represent a network of connected polymers. We also fix the unit mass m_u to the mass of a single bead. Hence, since the mass of a bacterium is usually $m \sim 1 \times 10^{-15}\,\mathrm{kg}$, this give us $m_u \sim 2.3 \times 10^{-18}\,\mathrm{kg}$. The time scale is so fixed to $\tau_{\mathrm{intrinsic}} = \sqrt{m_u l_u^2 / k_B T} \sim 5 \times 10^{-6}\,\mathrm{s}$.

During the biofilm growth, the bacteria produce polymers which cross link each other in a network, to which adhere the bacteria themselves. We reproduce these phenomenon by randomly placing some binding particles along the polymer chains, and on the external bacterium surfaces. First we equilibrate the system letting the polymer and the bacteria diffuse for roughly 4×10^6 time steps. Than, we randomly form from 100 to 500 polymer-polymer and polymer-bacterium crosslinks between non-bonded particles. The cross link is introduced with a new harmonic interaction $K_{CL}(r - \sigma)$, with $K_{CL} = 30$. The system is equilibrated again for other 4×10^6, and the procedure is repeated again, until the desired number of crosslinks is formed. In this way, we generate a series of biofilm configurations, with number of crosslinks spanning from 100 up to 5300.

For each cross-linked configuration, we apply an external oscillatory shear deformation along the X–Y plane by changing the box size with the relation $L = L_0 + A\sin(2\pi t/\tau)$, where A is the amplitude of the deformation, ranging from $A = 2.5$ (box deformation $\sim 8\%$) to $A = 36$ (box deformation $\sim 112\%$) [11]. The period τ of the external stress is fixed to $\tau = 2 \times 10^5$ (corresponding to 4×10^6 integration steps), which corresponds to a frequency of $1\,\mathrm{Hz}$. Any simulation has run at least for 4 complete oscillations of the external shear. The resulting in-phase stress σ' has been calculated by fitting the xy component of

the stress tensor with the relation [11]:

$$\sigma_{xy} = \sigma' \sin(2\pi t/\tau) + \sigma'' \cos(2\pi t/\tau). \quad (10)$$

In Figure S5 we show the stress-strain data for the static and shaking biofilm, for different number of crosslinks.

References

[1] Jacques Monod. The Growth of Bacterial Cultures. *Annual Review of Microbiology*, 3(1):371–394, 1949.

[2] T. M Smith and Robert Leo Smith. *Elements of ecology.* Pearson Benjamin Cummings, San Francisco, CA, 2009. OCLC: 241304684.

[3] Pushpita Ghosh, Jagannath Mondal, Eshel Ben-Jacob, and Herbert Levine. Mechanically-driven phase separation in a growing bacterial colony. *Proceedings of the National Academy of Sciences of the United States of America*, 112(17):E2166–E2173, April 2015.

[4] Morgan Delarue, Jrn Hartung, Carl Schreck, Pawel Gniewek, Lucy Hu, Stephan Herminghaus, and Oskar Hallatschek. Self-Driven Jamming in Growing Microbial Populations. *Nature physics*, 12(8):762–766, August 2016.

[5] Rafael D. Acemel, Fernando Govantes, and Alejandro Cuetos. Computer simulation study of early bacterial biofilm development. *Scientific Reports*, 8(1):1–9, March 2018.

[6] Jacinta C. Conrad, Maxsim L. Gibiansky, Fan Jin, Vernita D. Gordon, Dominick A. Motto, Margie A. Mathewson, Wiktor G. Stopka, Daria C. Zelasko, Joshua D. Shrout, and Gerard C. L. Wong. Flagella and pili-mediated near-surface single-cell motility mechanisms in *P. aeruginosa*. *Biophysical Journal*, 100(7):1608–1616, April 2011.

[7] Jay A. Stotsky, Jason F. Hammond, Leonid Pavlovsky, Elizabeth J. Stewart, John G. Younger, Michael J. Solomon, and David M. Bortz. Variable Viscosity and Density Biofilm Simulations Using an Immersed Boundary Method, Part II. *J. Comput. Phys.*, 317(C):204–222, July 2016.

[8] Ya Liu and Anna C. Balazs. Modeling Biofilm Formation on Dynamically Reconfigurable Composite Surfaces. *Langmuir: the ACS journal of surfaces and colloids*, 34(4):1807–1816, 2018.

[9] Zhijie Xu, Paul Meakin, Alexandre Tartakovsky, and Timothy D. Scheibe. Dissipative-particle-dynamics model of biofilm growth. *Physical Review E*, 83(6):066702, June 2011.

[10] Robert D. Groot and Patrick B. Warren. Dissipative particle dynamics: Bridging the gap between atomistic and mesoscopic simulation. *The Journal of Chemical Physics*, 107(11):4423–4435, September 1997.

[11] Guido Raos, Margherita Moreno, and Stefano Elli. Computational experiments on filled rubber viscoelasticity: What is the role of particle-particle interactions? *Macromolecules*, 39(19):6744–6751, September 2006.
